# Identification of coronary calcifications in optical coherence tomography imaging using deep learning

**DOI:** 10.1038/s41598-021-90525-8

**Published:** 2021-05-28

**Authors:** Yarden Avital, Akiva Madar, Shlomi Arnon, Edward Koifman

**Affiliations:** 1 Electrical and Computer Engineering, Ben-Gurion University of the Negev, 8410501 Beer-Sheva, Israel; 2 Faculty of Health Sciences, Ben-Gurion University of the Negev, 8410501 Beer-Sheva, Israel; 3 Heart Institute, Soroka Medical Center, 8410101 Beer-Sheva, Israel

**Keywords:** Interventional cardiology, Data processing, Image processing, Machine learning

## Abstract

Coronary calcifications are an obstacle for successful percutaneous treatment of coronary artery disease patients. The optimal method for delineating calcifications extent is coronary optical coherence tomography (OCT). To identify calcification on OCT and subsequently tailor the appropriate treatment, requires expertise in both image acquisition and interpretation. Image acquisition consists from system calibration, blood clearance by a contrast agent along with synchronization of the pullback process. Accurate interpretation demands careful review by the operator of a segment of 50–75 mm of the coronary vessel at steps of 5–10 frames per mm accounting for 375–540 images in each OCT run, which is time consuming and necessitates some expertise in OCT analysis. In this paper we developed a new deep learning algorithm to assist the physician to identify and quantify coronary calcifications promptly, efficiently and accurately. Our algorithm achieves an accuracy of 0.9903 ± 0.009 over the test set at size of 1500 frames and even managed to find calcifications that were not recognized manually by the physician. For the best knowledge of the authors our algorithm achieves high accuracy which was never achieved in the past.

## Introduction

Coronary calcifications pose a challenge to the interventional cardiologist when performing percutaneous coronary intervention (PCI) as such lesions portend increased risk for complications, lower success rate and higher frequency of stent failure at follow-up^1^. Optical coherence tomography (OCT) is an advanced intracoronary imaging technique that allows high-resolution of the coronary structure including vessel lumen size and vessel wall characteristics up to the level of a single cell. OCT allows identification and quantification of the amount and location of calcification, plaque characteristics, vessel wall dissection and intraluminal thrombus^2–4^. In addition, OCT enables assessment and optimization of stent deployment including treatment of under-expansion and edge dissections^5–7^. Moreover, recently, an entity of calcific nodule was shown to be the etiology of acute coronary syndrome in a significant number of patients with higher rate of adverse events when treated percutaneously^8,9^. The advantage of OCT in coronary calcification quantification has led to a scoring system to predict stent under expansion and consequently stent failure^10^.

However, currently these OCT features are interpreted manually by the physician. It requires experience in image acquisition and interpretation and thereby interfering with the catheterization laboratory workflow. Therefore, an automatic algorithm for identify the coronary calcifications in the blood vessels is desirable. An algorithm that is based on deep learning (DL) can help achieve accurate automatic segmentation of calcific vessel segments. One of the main advantages of such algorithm is the automation of OCT images interpretation along with reduction in procedural overall time and subsequently reduction in cost.

Our aim was to develop such algorithm that can accurately perform these tasks. In this manuscript we will describe the methods used including image pre-processing and DL model building in order to perform automated segmentation with a quantitative image assessment results based on criteria of loss function, accuracy function and dice coefficient.

## Methods

In order to segment the calcifications on OCT images, we used methods from the fields of image processing and DL. We have built a plan that includes pre-processing the data, performing segmentation of the images manually and building a model based on deep learning in order to perform automated segmentation.

### Pre-processing the data

The OCT runs usually contain additional information beyond the area of interest. For example, in many OCT images there are parts that are irrelevant for the purpose of training a segmentation model such as areas where blood is not properly cleared by the contrast agent, and subsequently interferes with the optical rays transmitted through the OCT catheter, thereby it obscures the coronary lumen and vessel wall and hampers the quality of the image. In other cases, the OCT catheter which is pulled back automatically enters the guide catheter at the origin of the coronary vessel and no longer images the artery itself. Therefore, frames of inadequate quality were filtered out, leaving-in the most relevant frames in order to train a model efficiently. In addition, the OCT results contain other measurement which are irrelevant to the current purpose and therefore were also removed leaving only the raw images for the training process. In order, to augment the data, each image was rotated by 90°, 180° and 270°.

### Creating ground truth database

**Figure 1:**
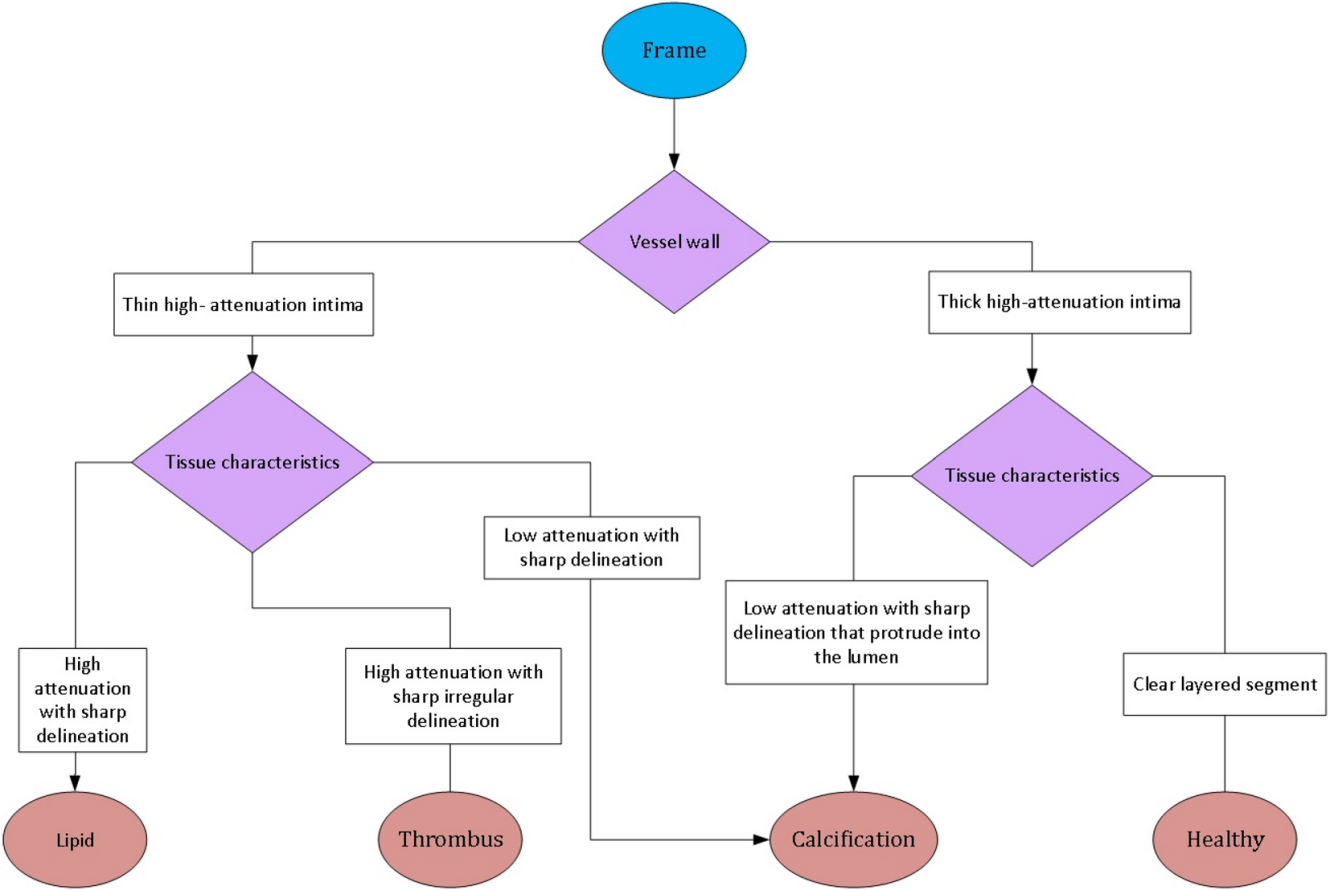
Identification of calcified segments flow chart. (Region of interest—vessel wall characteristics, symmetricity, irregularities etc.).

In order to be able to train a supervised deep learning model we are required to point towards the desired results apart from OCT imaging results. This is introduced into the model by the investigators in order to enable the learning process. For this purpose, Manual segmentation of calcifications in 8000 OCT images was performed by two independent individuals, from consecutive patients that underwent coronary angiography with OCT at Soroka Medical Center, Beer Sheva, Israel. Local ethics committee approved the present study and informed consent was waived due to the anonymization of the retrospectively collected data. In case of disagreement, a consensus of two annotators together with an expert interventional cardiologist was sought. This enabled to refine the data and correct mistakes in calcification identification. We expected that as we introduce more annotated data the results of the training will become more robust and its performance will improve over the test set. The process of manual segmentation was conducted according to the current consensus and described in Fig. 1^11^.

**Figure 2:**
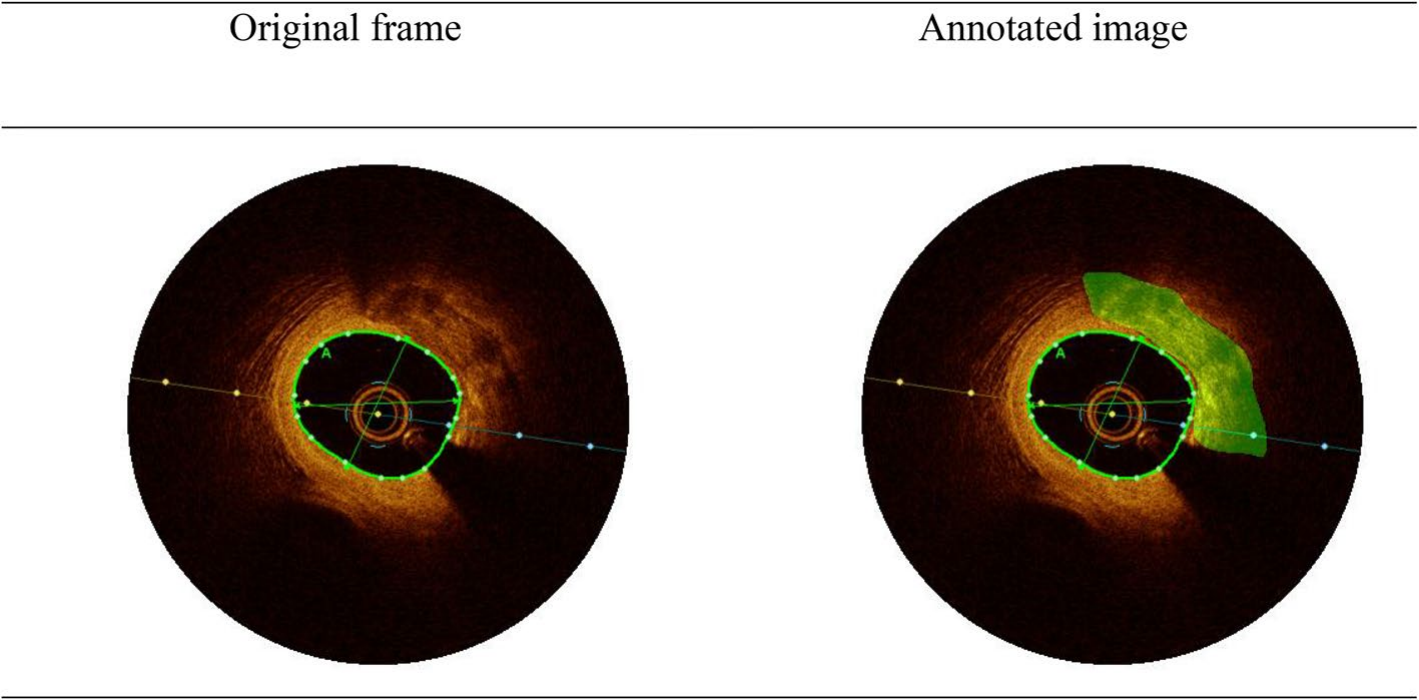
Example of single frame in the data set we created. The calcified area in the coronary is marked with green in the annotated image.

Each frame is described by three layers that corresponds to RGB colors represented as a three-dimensional tensor with size of (400, 400, 3). For each frame we created a mask that represents the area where calcification occurs. This mask is a matrix with size of (400, 400) the mask consists of arrays of 0 and 1 where 1 represents a pixel where there is calcification and 0 represents a pixel without calcification. We organized and sorted all the frames and their masks in order to provide the highest quality data for the model. We carefully selected the frames with the best accuracy of manual segmentation and dropped frames with equivocal findings (Fig. 2). We also selected for the training process only frames in which calcification was found. In addition, in cases where we discovered that there was a mistake in the human identification of the calcification, we corrected the masks of those frames. After filtering the data by removing corrupted frames, wrong annotations and frames without segmentation at all, we were left with only 540 frames for the purpose of training and testing the model. We divided the data to two sets, 490 of them used for training and 50 frames for the purpose of testing the model.

### CNN Model for segmentation

Following a review of the U-net architecture^12^, we noted that it can provide adequate results in the field of medical image segmentation^13^. We modified the model structure so that it could receive as input OCT data in large size (400, 400), and we also modified the size of the filters to fit larger images than the images inserted into the model in the original article^12^. Resizing the filters allows the model to be able to distinguish objects that are spread over more than (3, 3) pixels, so we increased the size of the filters in the down-sampling process to (5, 5) filters. In addition, we changed the size of the model output to (400, 400) to match the size of the frames inserted into the model.

### Model description

**Figure 3:**
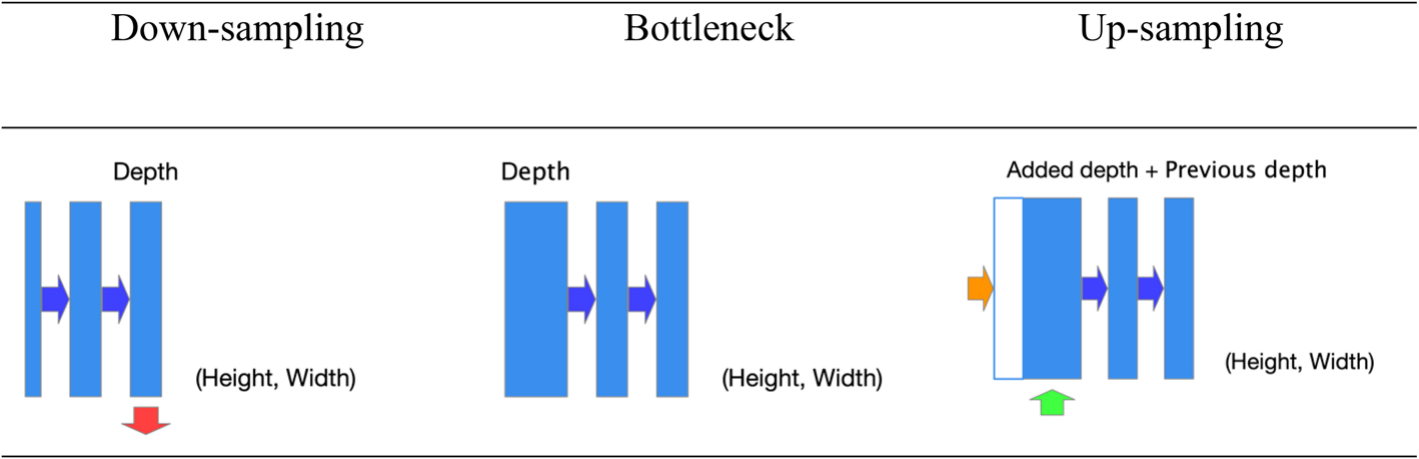
Visual block description. blue arrow stands for convolution layer operation, red arrow stands for Max pooling operation, green arrow stands for up-sampling with zero padding and blue boxes describe the size of the feature maps.

The architecture of the model we implemented is based on a convolution neural network and was designed to quickly and accurately segment medical images. The model is built symmetrically, which means that it is easier to unify data coming from different layers in the network. In this network, features are collected during the down-sampling blocks and concatenating to the data enters the up-sampling blocks, this operation helps keep the location which the features came from in the 2D image space (Fig. 3).

### Block description

- The down-sampling block extracts features and reduces the dimensions of the data entering it, this block is made up of an input layer, which passes through two convolution layers and then a max pooling operation is performed which reduces the dimensions of the data and produces the output of the down-sampling block.
- The bottleneck block extracts features and preserves the dimensions of the data entering it, this block is made up of an input layer, this layer passes through two layers of convolution that produce the output of the bottleneck block.
- The up-sampling block receives data from a layer with smaller data dimensions and increases its dimensions. Then it attaches data coming from parallel layers in the network, and extracts features from this data. The block consists of an up-sampling operation, a concatenating operation with data from another layer which then the passess through two convolution layers to produce the output of the up-sampling block.

### Model architecture

Our model consists of four down-sampling blocks, one bottleneck block and four up-sampling blocks.

The blocks are connected one after the other as follows.

**Figure 4:**
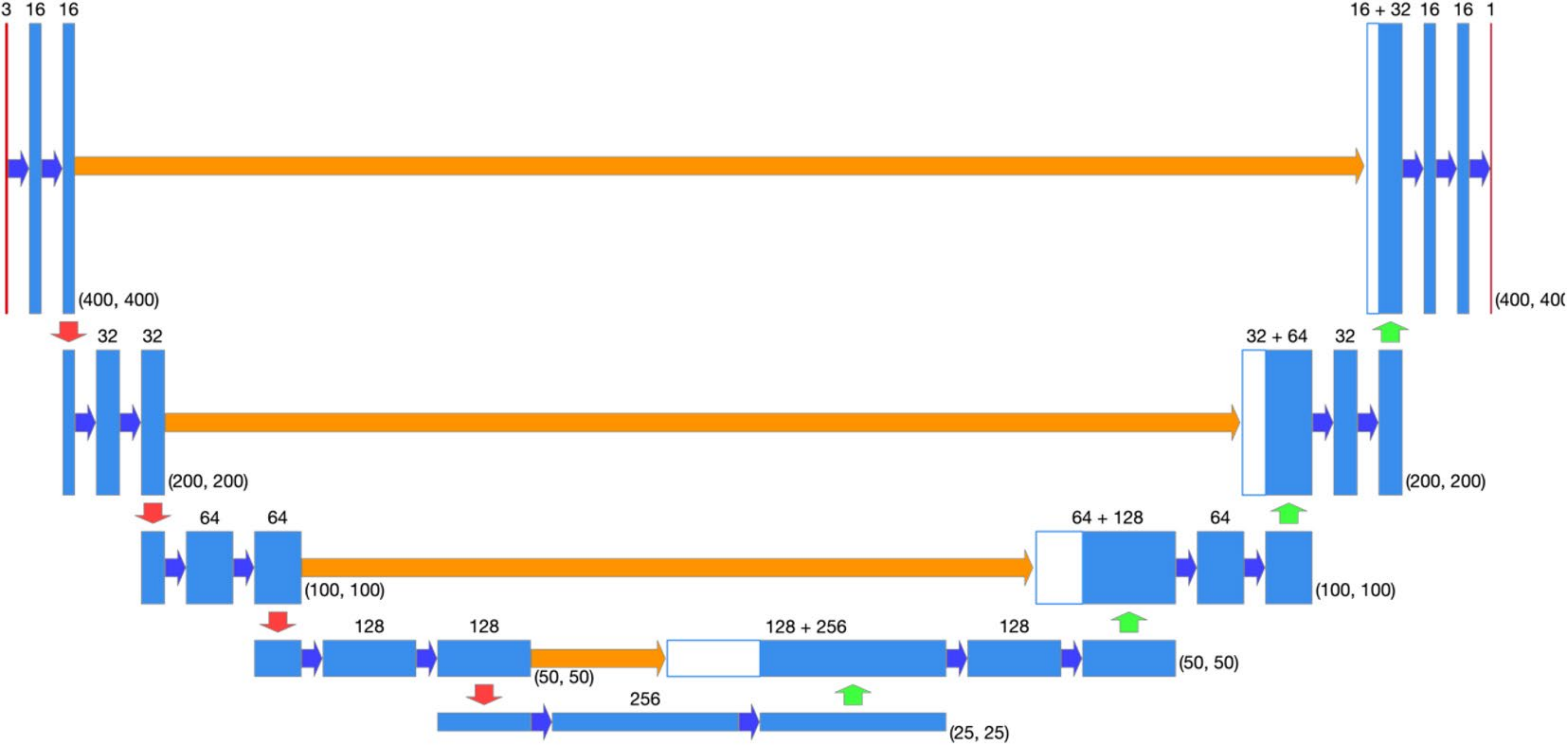
Deep learning Model architecture described by the fundamental blocks.

A single RGB frame is the entrance layer of the model, this frame is described by three matrices with the size of (400, 400) pixels, the entrance layer passes through the first down-sampling block which produces 16 feature maps with size of (200, 200) pixels, then the features pass through three more down-sampling blocks which create 128 feature maps with size of (50, 50) pixels, these features go into a bottleneck block and from there the up-sampling process begins, the up-sampling process consists of four up-sampling blocks. The feature maps that enters the up-sampling blocks collected from parallel blocks that belong to the down-sampling process as well as feature maps from the previous block. Upon completion of the up-sampling process, 16 feature maps with size of (400, 400) pixels are obtained. In order to produce the output layer we add one more convolution layer with kernel size of (1, 1) and sigmoid activation function in order to receive a single output frame with values from 0 to 1 (Fig. 4). This was constructed in order to provide the operator the ability to fine tune the system as a probability based.

## Results

In order to evaluate the quality and accuracy of calcification segmentation, we defined two main criteria. The first is a qualitative criterion by visual assessment of an interventional cardiologist. The second is a quantitative criterion measured by using loss function, accuracy function and dice coefficient with the data fed into the model in its original form without any coordinate transformation.

### Qualitative assessment

**Figure 5:**
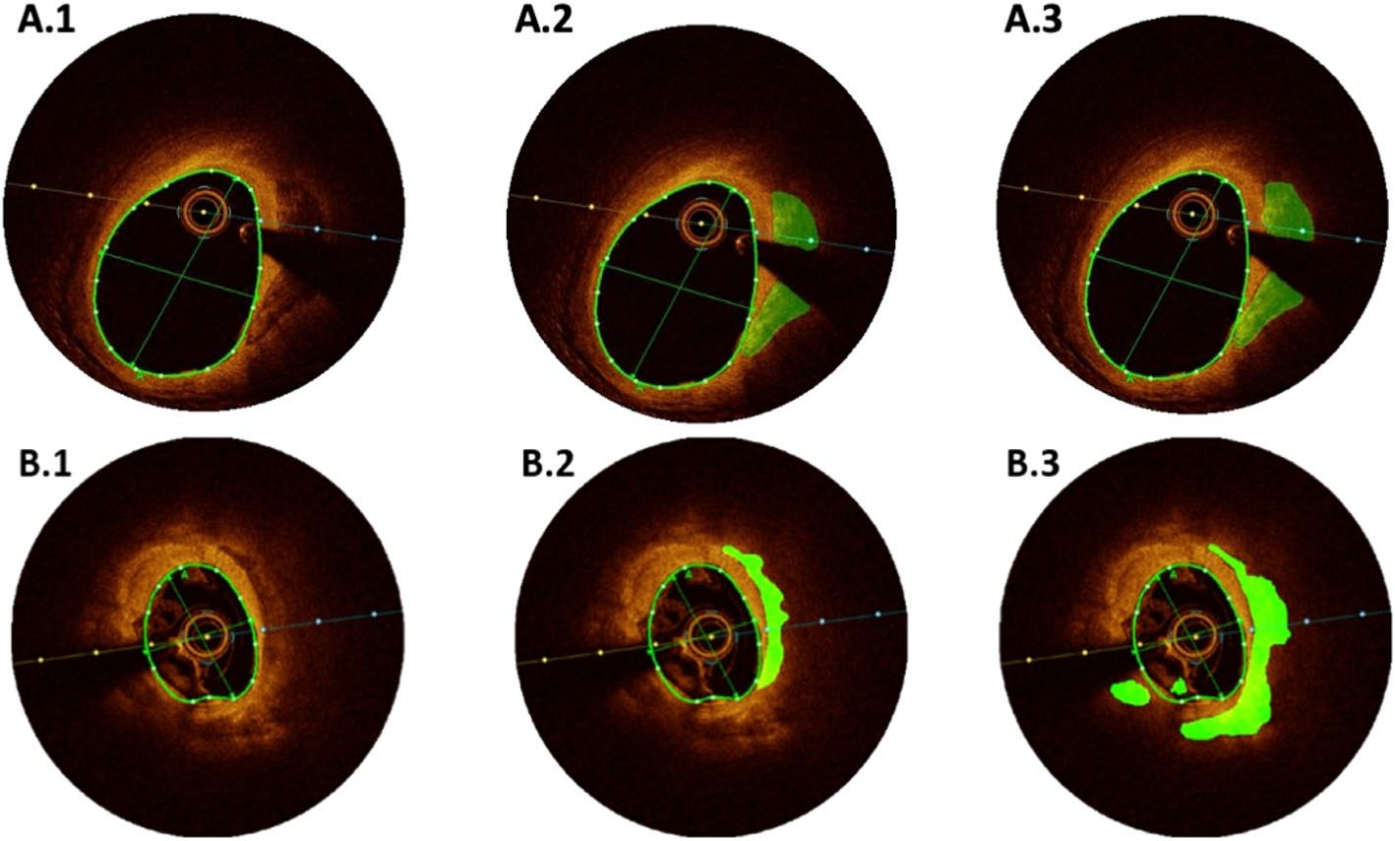
Visualize Results. Two examples of automated segmentation. (**A**) High accuracy of the model (**B**) Improved accuracy over manual annotation. (1) Original Image. (2) Manual annotation. (3) Automatic segmentation.

We compared the model prediction results to the manual annotation by analyzing the original image with the manual annotated image and the corresponding model image prediction. Approximately in 90% of the cases the cardiologist indicated that model prediction of calcification is correct. As a part of this test we noticed that in some cases the model result is more accurate than manual annotation, since some of the manual annotations had incomplete delineation of coronary calcifications. Example for two different situations of visual assessment are shown in Fig. 5. The green area in annotated images is the calcification area both prediction and manual annotation.

### Quantitative assessment

For a quantitative assessment of the quality of the model prediction results we used the next measures: accuracy loss function, dice coefficient metric and accuracy metric. The loss measure^14^ is calculated on a training and testing set, and its interpretation is based on how well the model is doing in these two sets. It is calculated as the mean of errors made for each image in training or testing sets. In the field of segmentation of medical images, the Binary Cross-Entropy function (1) has shown excellent results, therefore we decided to use it in our model as well. The concept of this function is that for each true calcification pixel (${\text{y}}_{\text{i}}=1$) it adds the log probability of calcification. Conversely, it adds the log probability of no calcification, for each true no calcification pixel (${\text{y}}_{\text{i}}=0$).

1 $${\text{Binary Cross - Entropy}} = {\text{H}}_{{\text{p}}} \left( {{\text{y}}_{{\text{i}}} ,{\text{p}}\left( {{\text{y}}_{{\text{i}}} } \right)} \right) = - \frac{1}{{\text{N}}}\sum\limits_{{{\text{i}} = 1}}^{{\text{N}}} {{\text{y}}_{{\text{i}}} } \log \left( {{\text{p}}\left( {{\text{y}}_{{\text{i}}} } \right)} \right) + \left( {1 - {\text{y}}_{{\text{i}}} } \right)\log \left( {1 - {\text{p}}\left( {{\text{y}}_{{\text{i}}} } \right)} \right)$$

An accuracy metric (2)^15^ is used to measure the model’s performance in an interpretable way. The accuracy of the model is a measure of how many both True Positive (TP) pixels and True Negative (TN) pixels exist in each image. It is the measure of how accurate the model's prediction is compared to the true data. Our optimize model scored $0.9903 \pm 0.009$ in this parameter.

2 $$\text{Accuracy}=\frac{\text{TP}+\text{TN}}{\text{TP}+\text{TN}+\text{FP}+\text{FN}}$$

A dice coefficient metric (3) is^16^ a measure of how many TP exist in each image. This metric penalizes for the false positives (FP) and false negative (FN) that the method finds. Unlike accuracy index this metric is not affected by the relative size of the calcification in the image. Our optimized model scored $0.7143\pm 0.2609$ in this parameter.

3 $${\text{D}}.{\text{C}} = \frac{{2\left| {{\text{X}} \cap {\text{Y}}} \right|}}{{\left| {\text{X}} \right| + \left| {\text{Y}} \right|}};\quad \left| {\text{A}} \right| = {\text{~}}\mathop \sum \limits_{{{\text{a}}_{{\text{i}}} \in {\text{A}}}} {\text{a}}_{{\text{i}}} ;\quad \left( {{\text{A}} \cap {\text{B}}} \right)_{{\text{i}}} = {\text{A}}_{{\text{i}}} \cdot {\text{B}}_{{\text{i}}} {\text{~}}$$

**Figure 6:**
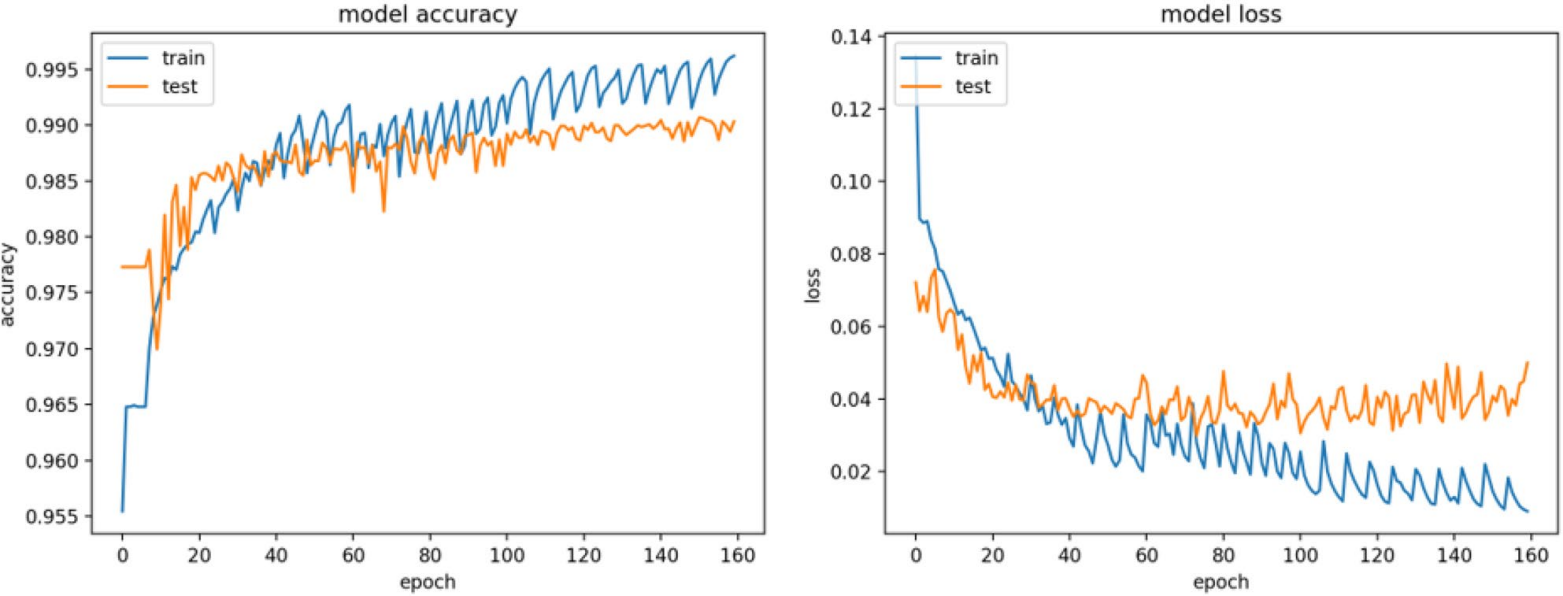
Accuracy and loss values throughout the learning process. x-axis show the number of epochs, y-axis show accuracy and loss values respectively.

The results of accuracy and dice coefficient metrices are considered a significant achievement in relation to the results of similar studies^17^. Figure 6 shows the learning process of the model over training and testing data in terms of accuracy and loss measures.

The graphs show also that the model learns very well in the first 100 epochs (sharp slope). Then the pattern of testing set graph remains approximately constant while the graph of the training set continues to improve. This process shows that the model is about to become over-fitted and therefore finished learning and from the 100th epoch the model begins to learn more specific details about the training set, which is irrelevant in our case because the model should be implemented on new data.

In addition, the pattern of training graph both in loss and accuracy functions resembles saw-tooth wave, the reason is that the training stage consists of 30 iterations, when each iteration of the model is fitted with altered data five times which is the epochs number. While the altered data is produced by rotation and permutation.

## Discussion

In this paper, we have shown that using algorithms from the field of DL and high-quality data, it’s possible to train a model that aims to perform automatic segmentation of calcifications in coronary arteries on top of products of OCT technology, with very high accuracy. Our algorithm supports interventional cardiologists decisions when performing PCI in calcified coronary arteries. The algorithm could help achieve better success rates and long-term clinical outcome for the patients.

When we started the initial training stages of the model, our results had lower quality than the final result. After an in-depth review of the results we noticed that the model was able to differentiate areas with and without calcifications, but we are unable to accurately find the boundary between a healthy area and an unhealthy area. Following these results, we decided to perform a massive sifting of the data for training, we discovered that there are samples where the manual segmentation we performed was not accurate enough. Following exclusion of low-quality samples, we performed the model training process again. This process now included only 490 frames, compared to 1500 frames before filtering, resulting in a significant improvement in the dice index which rose from a score of 0.47–0.65. On the same architecture of model. This improvement indicates a very high sensitivity of the model to the data quality. Since we observed that the quality is superior to the quantity and we decided to continue with the sifting data set where 490 frames were used for training and only 50 frames were used for testing.

Another significant improvement we were able to achieve during the study concerns the traditional structure of the U-net network. Our main task in this study was to identify the calcifications on OCT image. In most cases despite the high focus and resolution of the technology, the calcifications sometimes have unclear boundaries which makes it difficult to discern their borders. This phenomenon forced us to work at a relatively high resolution compared to what is accepted in the field of deep learning (400, 400). In order to distinguish details within an image at such a high resolution, filters larger than (3, 3) are needed, because on the order of 9 pixels, it is almost impossible to distinguish different trends in the image, nor can the textures of a diseased or healthy area be discerned. Following this distinction, we changed the size of the filters in the down-sampling process to size filters (5, 5) which actually gave us a much better ability to notice the different trends. Finally, in order to increase the accuracy towards the network output we left the filters of the up-sampling and bottleneck process at the size of (3, 3) in order to focus the decision of the network to smaller areas. Subsequently, we noted another significant improvement in the results that led to the final results of 0.71 in the dice index.

Further research that could lead to further improvements in the identification quality of the calcifications would include a DL model based on three-dimensional convolution in order to allow the model to diagnose the immediate environment of each frame and thereby improve the predictability for each frame. This can be achieved for example by building a model that takes three frames at a time and performs a three-dimensional convolution with size filters (5, 5, 3) to test feasibility. In addition, the effect of pre-identification of the lumen of the artery should be investigated and the results of identifying the artery lumen provided as an additional input to the model identifying the calcifications because in many cases the calcifications distort the shape of the artery and early detection of this deformity can help identify the calcification.

## Conclusions

DL algorithm can successfully and accurately identify coronary calcification on OCT images in an automated manner. This may potentially assist the interventional cardiologist to apply the appropriate measures in treating calcified lesions and consequently achieve optimal procedural success and therefore improved long-term result. Further algorithm improvement with more data, and additional features will result in superior results. Future research should consider other types of network such as U Net 3+^18^, meanwhile the present results already provide the operator a useful tool that may be very valuable.
